# Bushen Huoxue Attenuates Diabetes-Induced Cognitive Impairment by Improvement of Cerebral Microcirculation: Involvement of RhoA/ROCK/moesin and Src Signaling Pathways

**DOI:** 10.3389/fphys.2018.00527

**Published:** 2018-05-15

**Authors:** Yuan Li, Quan Li, Chun-Shui Pan, Li Yan, Bai-He Hu, Yu-Ying Liu, Lei Yang, Ping Huang, Shao-Yang Zhao, Chuan-She Wang, Jing-Yu Fan, Xue-Mei Wang, Jing-Yan Han

**Affiliations:** ^1^Integrated Laboratory of Traditional Chinese Medicine and Western Medicine, Peking University First Hospital, Beijing, China; ^2^Tasly Microcirculation Research Center, Peking University Health Science Center, Beijing, China; ^3^Key Laboratory of Microcirculation, State Administration of Traditional Chinese Medicine of China, Beijing, China; ^4^Key Laboratory of Stasis and Phlegm, State Administration of Traditional Chinese Medicine, Beijing, China; ^5^Department of Anatomy, Peking University Health Science Center, Beijing, China; ^6^Department of Integration of Chinese and Western Medicine, School of Basic Medical Sciences, Peking University, Beijing, China

**Keywords:** diabetes-induced cognitive impairment, bushen huoxue prescription, neuron damage, AGEs/RAGE/RhoA, Src

## Abstract

Type 2 Diabetes mellitus (T2DM) is closely correlated with cognitive impairment and neurodegenerative disease. Bushen Huoxue (BSHX) is a compound Chinese medicine used clinically to treat diabetes-induced cognitive impairment. However, its underlying mechanisms remain unclear. In the present study, KKAy mice, a genetic model of type 2 diabetes with obesity and insulin resistant hyperglycemia, received a daily administration of BSHX for 12 weeks. Blood glucose was measured every 4 weeks. After 12 weeks, BSHX treatment significantly ameliorated the T2DM related insults, including the increased blood glucose, the impaired spatial memory, decreased cerebral blood flow (CBF), occurrence of albumin leakage, leukocyte adhesion and opening capillary rarefaction. Meanwhile, the downregulation of the tight junction proteins (TJ) claudin-5, occludin, zonula occluden-1 (ZO-1) and JAM-1 between endothelial cells, amyloid-β (Aβ) accumulation in hippocampus, increased AGEs and RAGE, and expression of RhoA/ROCK/moesin signaling pathway and phosphorylation of Src kinase in KKAy mice were significantly protected by BSHX treatment. These results indicate that the protective effect of BSHX on T2DM-induced cognitive impairment involves regulation of RhoA/ROCK1/moesin signaling pathway and phosphorylation of Src kinase.

## Introduction

Diabetes mellitus (DM) is a chronic metabolic disorder, which affects at least 382 million people worldwide (Roy et al., 2015). DM is characterized by high rate of mortality and morbidity due to numerous complications, of which cognitive dysfunction has been considered the most prevalent and significant one, especially in old people with type 2 diabetes mellitus (T2DM) (Strachan, 2011; Talbot et al., 2012; Prasad et al., 2014; Ascher-Svanum et al., 2015; Fried et al., 2016; Chen et al., 2017). The etiology of cognitive impairment is not completely identified, albeit, the impact of T2DM on vasculature and neuron is currently thought to contribute to the development of cognitive impairment (Zlokovic, 2008; Greenberg et al., 2009; Viswanathan et al., 2009; Quaegebeur et al., 2011; Iadecola, 2013; Li et al., 2016). Advanced glycation end-products (AGEs), irreversible adducts of the Maillard reaction, are known to be involved in the pathogenesis and prognosis of diabetic microvascular complications and Alzheimer's disease (Yamagishi et al., 2015; Zhang et al., 2017). RAGE, the membrane receptor for AGEs, is also implicated in diabetes complications including the injury of the brain (Yamagishi et al., 2015). Activation of the AGEs–RAGE axis evokes inflammatory responses through several signaling pathways, resulting in endothelial dysfunction, among which the RhoA/ROCK/moesin pathway plays a critical role in vascular barrier dysfunction (Wang et al., 2012, 2016). Additionally, AGEs-induced Src phosphorylation and increase of Aβ has been implicated in tight junction loss, leading to endothelial barrier dysfunction (Basuroy et al., 2003; Adam et al., 2016; Chen et al., 2016; Spampinato et al., 2017). Therefore, an approach that inhibits AGEs-RAGE induced activation of RhoA/ROCK/moesin signaling pathway is expected to attenuate the cerebral microangiopathy and thus prevent the cognition impairment in T2DM.

Bushen Huoxue (BSHX) is a newly formulated compound Chinese medicine containing 7 components, as listed in Table 1. Previous studies have shown the role of its main components in reducing blood glucose and improving cerebral microcirculatory disturbance and cognitive impairment (Zhou et al., 2002; Hong et al., 2007; Zeng et al., 2010; Yan et al., 2014). BSHX has been used clinically to improve diabetic dementia with efficiency. However, the underlying mechanisms remain unclear. We hypothesized that BSHX ameliorates diabetic dementia by inhibiting AGEs-RAGE expression and RhoA/ROCK/moesin signaling pathway. The aim of the present study was to test this hypothesis in a spontaneous T2DM animal model: KKAy mice.

**Table 1:** Composition of BSHX.

**Species**	**Chinese name**	**Plant part**	**Family**	**Percentage of total weight**
*Lycium barbarum* L	Gouqi	Fruit	Solanaceae	25
*Cuscuta chinensis* Lam	Tusizi	Seed	Convolvulus	25
*Schisandra chinensis* (Turcz.) Baill	Wuweizi	Fruit	Magnolia Branch	3
*Rubus chingii* Hu	Fupenzi	Fruit	Rosaceae	12
*Plantago asiatica* L	Cheqianzi	Seed	Vehicle Section	6
*Epimedium brevicornu* Maxim	Yinyanghuo	Stem Leaf	Berberidaceae	25
*Whitmania pigra* Whitman	Shuizhi	The whole animal	Hirudinidae	4

## Materials and methods

### Animals

KKAy mouse was produced by transferring the yellow obese gene (Ay allele) into the KK/Ta mouse, a glucose-intolerant black KK female mice. This animal has been widely used as a model of type 2 diabetes mellitus (Sakata et al., 2010; Tomino, 2012) with C57BL6J as the strain homology control mice (Iwatsuka et al., 1970; Herberg and Coleman, 1977). This study included 80 male KKAy mice and 20 C57BL/6 mice with body weight of 30 ± 5 g (Beijing HFK Bioscience Co., Ltd [License No. SCXK (Jing) 2014-0004]). All animals were housed in a regulated environment: temperature of 25 ± 2°C and humidity of 55 ± 5% with 12/12 h light/dark cycle for at least 1-week before experiment. The animals were fasted for 12 h prior to the experiment with water given *ad libitum*. All animal treatments in this study were conducted in accordance with international ethical guidelines concerning the care and use of laboratory animal, and approved by the Committee of Peking University First Hospital (Approval Number: J201534).

### BSHX

BSHX was manufactured as granules after dynamic cycle extraction and concentrated by evaporating and spray drying. The drugs were then packed with aluminum foil composite (3 g/bag).

### Animal treatment

After acclimation for 1 week, blood glucose was measured by the glucose oxidase method. The KKAy mice with random blood glucose ≥ 11.1 mmol/L or fasting blood glucose ≥ 7.0 mmol/L were recruited. The animals were randomly divided into 4 groups: C57 group, KKAy group, KKAy + BSHX 1 g/kg group, and KKAy + BSHX 2 g/kg group. These two doses of BSHX were selected based on our preliminary experiments. All drugs were dissolved in distilled water to a concentration of 100 mg/ml or 200 mg/ml, respectively, prior to administration. In the C57BL6J and KKAy groups, animals were given equivalent volume of distilled water in the same manner. All drugs were administered by oral gavage once per day for 12 weeks. Blood glucose was measured every 4 weeks.

### Morris water maze test

After 12 weeks of treatment, spatial and related forms of learning and memory were assessed by Morris Water Maze (MWM) as previously described (Song et al., 2015). Briefly, mice were individually trained in a circular pool (120 cm in diameter and 50 cm in height), which was filled with water to a depth of 30 cm and maintained at 25°C. A platform (9 cm in diameter) was located in the center of one quadrant of the pool. On each side of the walls of the four quadrants, distinct colored paper was disposed as a visual positional hint. On first 2 days, each mouse was subjected to visible-platform training, in which the platform was submerged 1 cm beneath the water surface and was indicated by a flag. On day 3–5, a hidden-platform training was performed by removal of the flag for spatial learning and memory retention in terms of the ability to find the platform.

The test of escape latency in finding the platform was then conducted for 5 days, in which each mouse received four trials per day with about 1 h interval in between. During each trial, mice were released into the water facing the pool wall at one of the 4 starting positions, and allowed to locate the submerged platform for a maximum of 120 s. If a mouse failed to find the platform within 120 s, it was gently guided to the platform and allowed to stay on it for 15 s. On day 6, the platform was removed and a probe trial was performed, in which the time spent in the target quadrant where the escape platform was placed was noted, and the number of crossings over the original position of the platform was recorded.

### Cerebral blood flow measurement

Mice treated with BSHX for 12 weeks were anesthetized with pentobarbital sodium (0.1 g/kg body weight) intraperitoneally, and cerebral blood flow (CBF) was measured using Laser Doppler perfusion image system (PeriScan PIM3 System; PERIMED, Stockholm, Sweden) as previously described with some modifications (Huang et al., 2012). Briefly, a computer-controlled optical scanner was applied with the scanner head maintaining in parallel to the cerebral cortex surface at a distance of 18 cm, which directed a low-powered He-Ne laser beam over the exposed cortex. As such, the tissue could be illuminated to a depth of 0.5 mm at each measuring point. A color-coded image denoting specific relative perfusion levels was displayed on a video monitor with blue to red representing low to high. The image was evaluated by the software LDPIwin 3.1.

### Microcirculation observation

A noninvasive method was applied to observe albumin leakage from cerebral venules as previously described (Sun et al., 2010). For this purpose, the animal's head was secured in a stereotactic frame, the skull was exposed through a midline incision, and ground down with a cranial drill over the right parie to occipital cortex. This location corresponds to the margin of the middle cerebral artery (MCA) supplied territory. Injuries in MCA territory could lead to diverse neurologic deficits including memory impairments (Bingham et al., 2012). Fluorescein FITC-albumin (Sigma-Aldrich, St Louis, MO, USA) was then infused (50 mg/kg body weight) through the femoral vein 10 min before observation. An upright fluorescence microscope (BX51WI, Olympus, Tokyo, Japan) was employed to acquire venular images under irradiation at wavelength of 488 nm. The cerebral venules ranging from 35 to 50 μm in diameter and 200 μm in length were selected. The fluorescence intensities of FITC-albumin inside the lumen of selected venule (Iv) and the surrounding interstitial area (Ii) were assessed with Image-Pro Plus 5.0 software. Albumin leakage was expressed as the ratio Ii/Iv (Huang et al., 2012).

For determination of adherent leucocytes to microvessels, the fluorescence tracer R6G (5 mg/kg body weight, Fluka Chemie AG, Switzerland) was injected into to the animal via the femoral vein 10 min before observation. The venule images were acquired under irradiation at wavelength of 543 nm. The leukocytes that attached to the venular wall for more than 30 s were defined as the adherent leukocytes. The number of adherent leukocytes was counted along venules and presented as the number per 200 μm of venule length (Sun et al., 2010).

### Tissue preparation and protocols for histology

Mice under anesthesia were infused via the left ventricle first with heparinized phosphate-buffered saline, followed by 4% formaldehyde in 0.1 M phosphate-buffered saline for 40 min for fixation. Brains were harvested and kept in the same fixative for 48 h. Then the brains were placed in 30% sucrose at 4°C for at least 2 days before being embedded in OCT (Miles Inc., Elkhart, IN, USA) and frozen in 2-methybutane cooled in liquid-nitrogen. Coronal frozen sections of 10 μm thick were sliced with a cryostat microtome (CM1900; Leica, Nussloch, Germany) at −20°C from the optic chiasma to the cerebral caudal end (Yan et al., 2014). The slices were processed for Nissl staining or immunohistochemistry staining after air dried.

To stain Nissl bodies in neurons, series sections were immersed in cresyl violet (Sigma Aldrich, St Louis, Missouri, USA) for 2 h at 37°C, followed by dehydration and hyalinization successively, and cover-slipped with mounting medium before observation.

To observe the density of opening capillaries, the sections were stained with mouse antibody against CD31 (Thermo Scientific, MA1-80069, Waltham, USA) overnight at the recommended concentration of 1:50 at 4°C. The samples were then incubated with a biotinylated secondary antibody followed by avidin-biotin-peroxidase complex. Positive staining was revealed with diaminobenzidine, and the nuclei were counterstained with haematoxylin. Five fields of CA1 sector in hippocampus of each animal were randomly selected, and images were captured by a digital camera connected to a microscope (BX512DP70, Olympus, Tokyo, Japan) and analyzed with Image-Pro Plus 5.0 software (IPP, Median Cybernetic, Bethesda, MD, USA). For evaluation of the expression of tight junction proteins in cerebral microvessels, immunofluorescence staining was performed. Slices were washed three times with PBS and blocked with 3% normal goat serum at room temperature for 0.5 h, followed by incubation with primary antibodies diluted in PBS overnight at 4°C. The primary antibodies applied included: mouse anti-claudin-5 (1:100, Invitrogen, Camarillo, CA, USA), mouse anti-occludin (1:50, Invitrogen, Camarillo, CA, USA), and rabbit anti-vWF (1:100, Millipore, Temecula, CA, USA). Following washing, the slices were processed by incubation with Dylight 488-labeled goat anti-rabbit IgG (KPL, Gaithersburg, MD, USA) and Dylight 549-labeled goat anti-mouse IgG (KPL, Gaithersburg, MD, USA) for 2 h at room temperature. The brain slices were further stained with Hoechst 33342 (Molecular Probes) to reveal the nuclei, and mounted, coverslipped, and photographed under a laser scanning confocal microscope (TCS SP5, Leica, Mannheim, Germany).

### Electron microscopy

The mouse brain was perfused for 50 min with 2.5% glutaraldehyde in 0.1 mol/L phosphate buffer at a speed of 3 mL/min. For transmission electron microscopy (TEM), a coronal slice of approximately 1 mm thick through the hippocampus area was taken. The slice was placed in freshly prepared 3% glutaraldehyde overnight at 4°C. Following treatment with 0.1 mol/L phosphate buffer for 3 times, the tissue block was post-fixed in 1% osmium tetroxide, dehydrated, then embedded in Epon 812. Ultrathin sections were prepared and examined in a transmission electron microscope (JEM 1,400 plus, JEOL, Tokyo, Japan). For scanning electron microscopy (SEM), the samples were cut into blocks and placed in the freshly prepared glutaraldehyde for 2 h, rinsed with 0.1 mol/L phosphate buffer, then post-fixed in 1% osmium tetroxide in 0.1 mol/L phosphate buffer for 2 h. The specimens were processed as routing and examined under a scanning electron microscope (JSM-5600LV, JEOL, Tokyo, Japan).

### Measurement of Aβ and AGEs

After 12 weeks of treatment, the blood and hippocampus tissues were collected from mice. The concentration of Aβ and AGEs in plasma and hippocampus tissues were determined by ELISA kits (Andy Gene Biotechnology Co., Ltd, Beijing, China) as per the manufacturers' instructions.

### Western blotting

Anesthetized mice were transcardially perfused with saline through the left ventricle. The brain was then removed and cerebral hippocampus was separated for extraction of protein. Protein concentrations were determined with a BCA Protein Assay Kit (Thermo Scientific). An equal amount of proteins was subjected to electrophoresis on SDS polyacrylamide gel, followed by electrotransfer to a nitrocellulose membrane. After blockage with 3% skimmed-milk powder in PBS with 0.1% tween-20 for 1 h, the membranes were probed overnight at 4°C with the primary antibodies specific for β-actin, claudin-5, occludin, ZO-1 (1:1000, Invitrogen, Camarillo, CA, USA), JAM-1 (1:200, Santa Cruz Biotechnology, Santa Cruz, USA), RAGE, RhoA, ROCK1, moesin, p-moesin (1:1,000, Abcam, Cambridge, UK), Src and p-Src (1:500, Cell Signaling, Beverly, Massachusetts, USA). After washing with TBST, the membranes were incubated with respective horseradish peroxidase-conjugated secondary antibodies (Beyotime, Shanghai, China) at a 1:3,000 dilution for 60 min at room temperature. Specific bands were visualized using enhanced chemiluminescence detection kit (Santa Cruz Biotechnology, California).

### Statistical analysis

All data were expressed as mean ± SD. Statistical differences were evaluated by analysis of one-way ANOVA followed by Tukey test or two-way ANOVA followed by Bonferroni for multiple comparisons (escape latency during 5 days of training). A probability < 0.05 was considered to be statistically significant.

## Results

### BSHX prescription reduces blood glucose in KKAy mice

**Figure 1:**
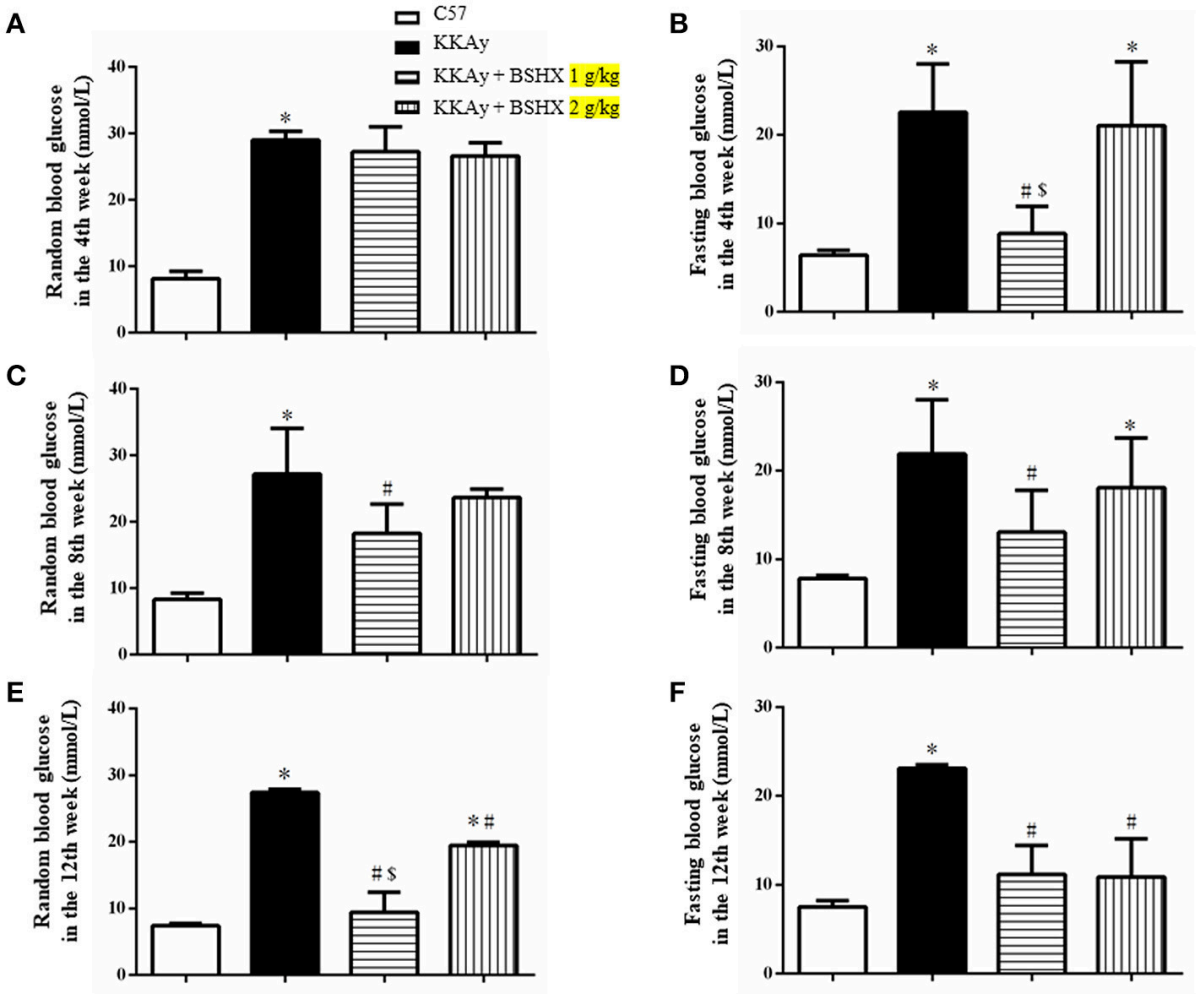
The effect of BSHX on blood glucose levels. Treatment of KKAy mice with BSHX for 4 **(A,B)**, 8 **(C,D)**, and 12 weeks **(E,F)** reduced random and fasting levels of blood glucose. Data were expressed as mean ± SD (*n* = 6). ^*^p < 0.05 vs. C57 group, ^#^*p* < 0.05 vs. KKAy group, ^$^*p* < 0.05 vs. KKAy+BSHX 2 g/kg group.

DM is a metabolic disease characterized by a high concentration of blood glucose. Thus, glucose levels of KKAy mice were examined every 4 weeks. We found that BSHX has no obvious effect on random blood glucose in the 4th week, whereas 1 g/kg BSHX significantly reduced the elevated fasting blood glucose after 4 weeks treatment (Figures 1A,B). The beneficial effect on both random and fasting blood glucose levels was observed in the 8th week (Figures 1C,D). Figures 1E,F revealed that BSHX prescription significantly prevented the elevation in levels of random blood glucose of KKAy mice after 12 weeks treatment with 1 g/kg BSHX being more effective compared with BSHX at the dose of 2 g/kg (*p* < 0.05). Besides, BSHX at both doses used remarkably prevented the increase in fasting blood glucose of KKAy mice with equal effectiveness.

### BSHX attenuates memory impairment in KKAy mice

**Figure 2:**
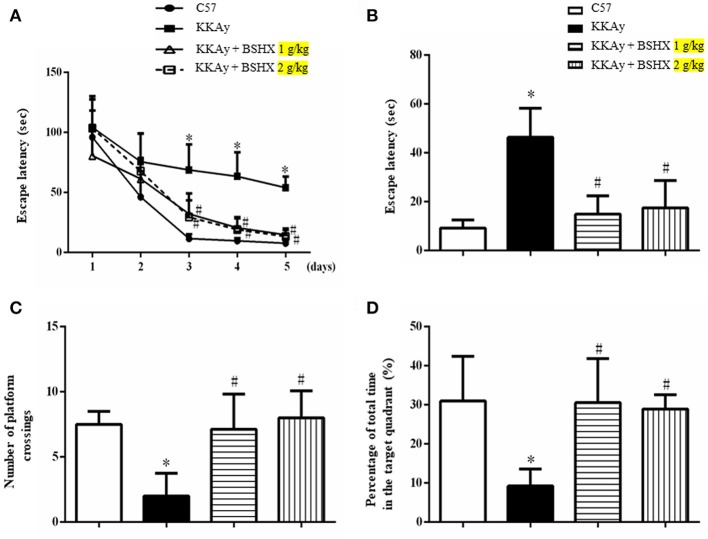
The effect of BSHX on spatial memory of mice. **(A)** The mean latency time during the training period. **(B)** The latency to platform in the probe trial. **(C)** The number of platform crossings during the spatial probe test. **(D)** The percent of total time spent in the target of quadrant during the spatial probe test. Data were expressed as mean ± SD (*n* = 6). ^*^p < 0.05 vs. C57 group, ^#^*p* < 0.05 vs. KKAy group.

MWM test was carried out to examine the spatial and related forms of learning and memory of mice. The escape latency gradually decreased in all groups over 5 days of training (Figure 2A). This decrease became significantly slower in KKAy group than that in C57 group starting from the third day, which was accelerated by treatment with BSHX at both doses reaching to a level close to C57 on day 5. The effect of BSHX in the improvement of memory impairment was further confirmed by the escape latency time tested on day 6, as displayed in Figure 2B. In the probe trial, a putative measurement of spatial learning and memory retention, mice in KKAy group displayed a significant decrease in the number of platform crossings compared with C57 groups (Figure 2C) and the percentage of total time in the target quadrant (Figure 2D). Treatment with BSHX remarkably increased the aforementioned index (Figure 2C).

### BSHX ameliorates CBF in KKAy mice

**Figure 3:**
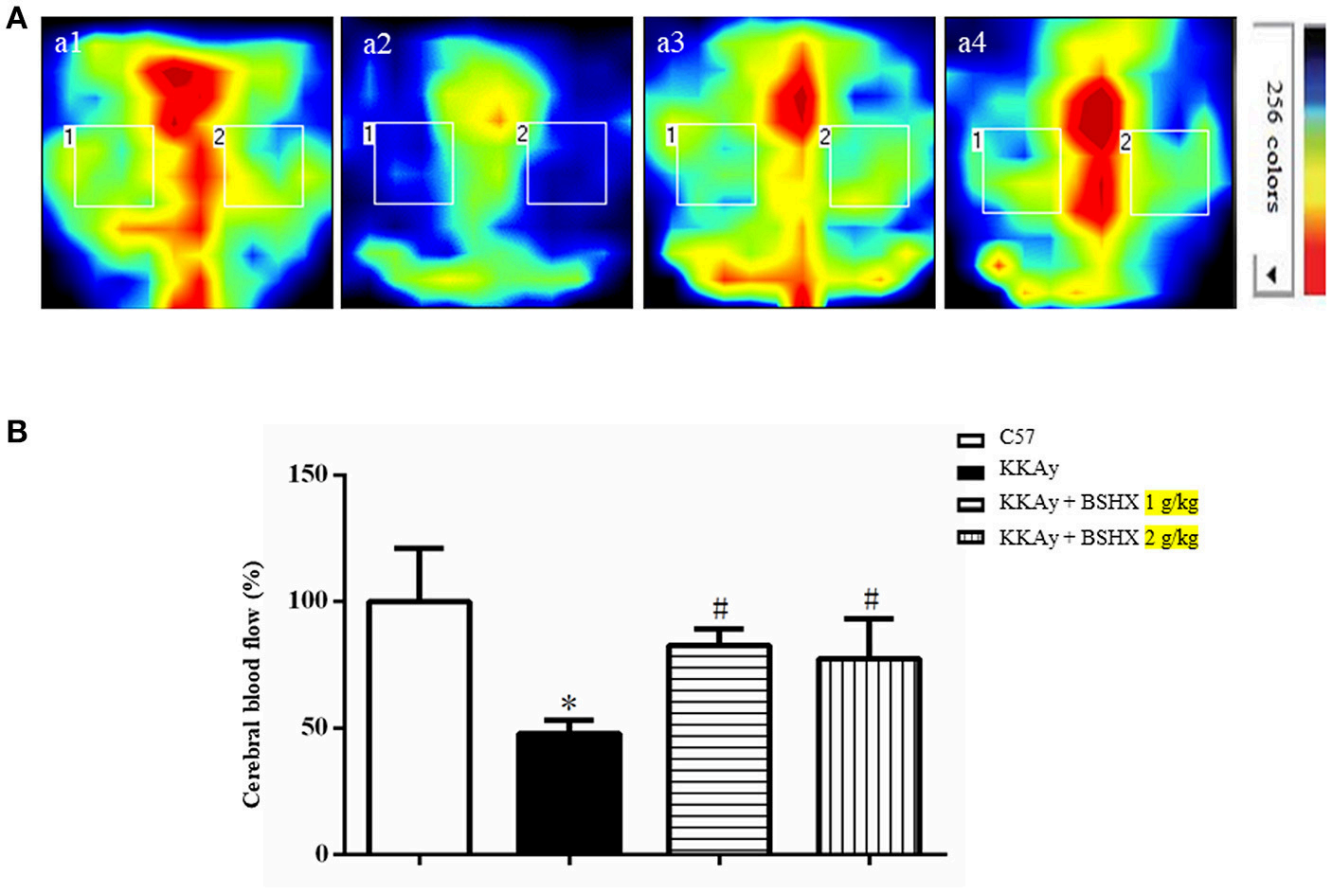
The effect of BSHX on CBF in mouse cerebral cortex. **(A)** Representative images of CBF of cerebral cortex in different groups. The magnitude of CBF is represented by different colors, with blue to red indicating low to high. a1: C57 group; a2: KKAy group; a3: KKAy + BSHX 1 g/kg group; a4: KKAy + BSHX 2 g/kg. **(B)** Quantitative analysis of CBF in all groups. CBF was determined by the average of the square box 1 and 2, which correspond to MCA territory. Data were expressed as mean ± SD (*n* = 6). ^*^p < 0.05 vs. C57 group, ^#^*p* < 0.05 vs. KKAy group.

CBF was determined by a laser Doppler perfusion image system post 12 weeks of treatment. Representative images and statistical analysis results were presented in Figures 3AB respectively. Impressively, a significant reduction in CBF of KKAy mice was observed compared with control group. BSHX notably improved CBF in KKAy mice.

### BSHX reduces albumin leakage and leukocyte adhesion in KKAy mice

**Figure 4:**
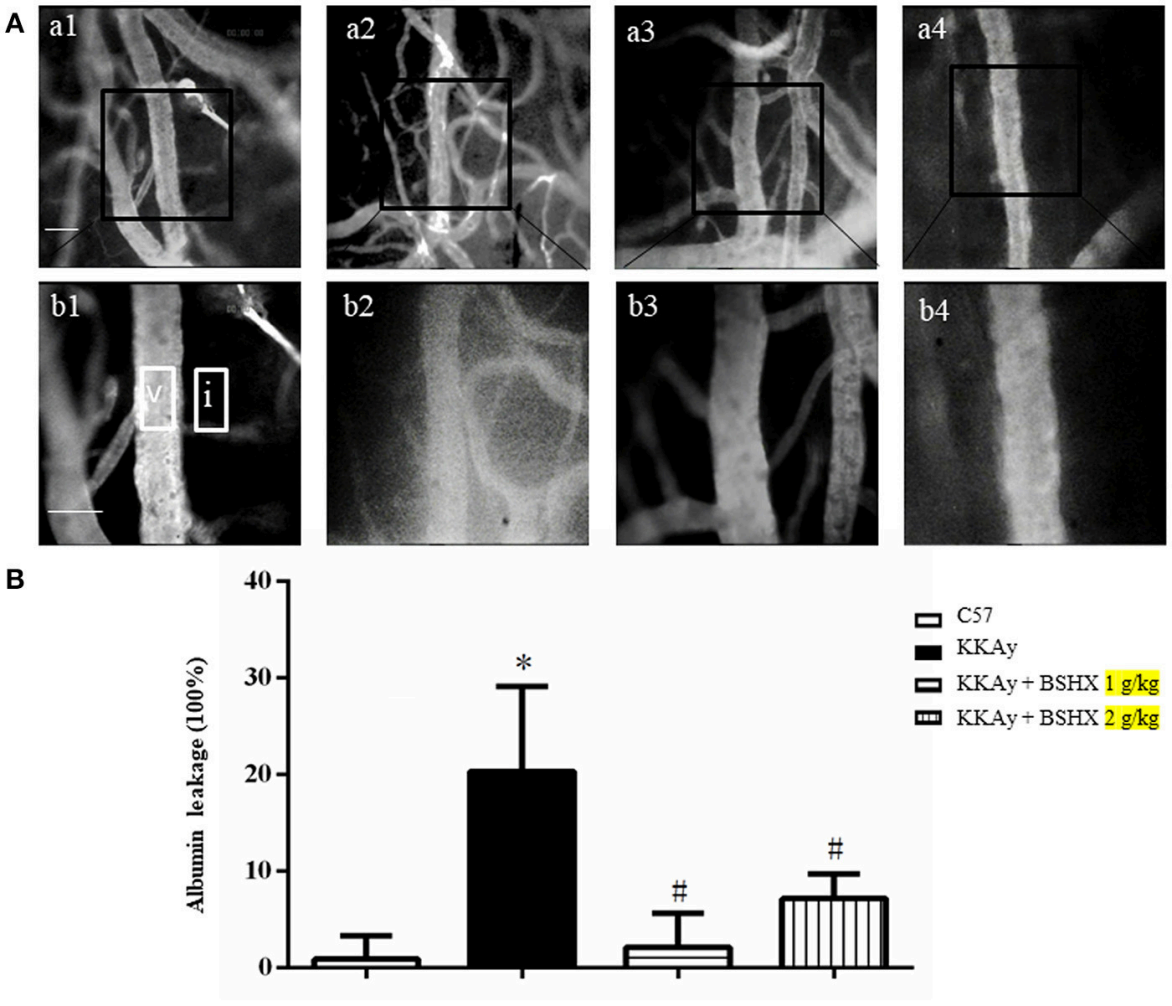
The effect of BSHX on albumin leakage from cerebral venules. **(A)** Representative images of albumin leakage from venules in all groups. Rectangles represent the areas for determination of fluorescence. V: cerebral venule. I: Interstitial tissue. a1: C57 group; a2: KKAy group; a3: KKAy + BSHX 1 g/kg group; a4: KKAy + BSHX 2 g/kg. High magnifications of a1–a4 are shown below as b1–b4, respectively. Bar = 50 μm. **(B)** Statistic analysis of albumin leakage. Data were expressed as mean ± SD (*n* = 6). ^*^p < 0.05 vs. C57 group, ^#^*p* < 0.05 vs. KKAy group.

**Figure 5:**
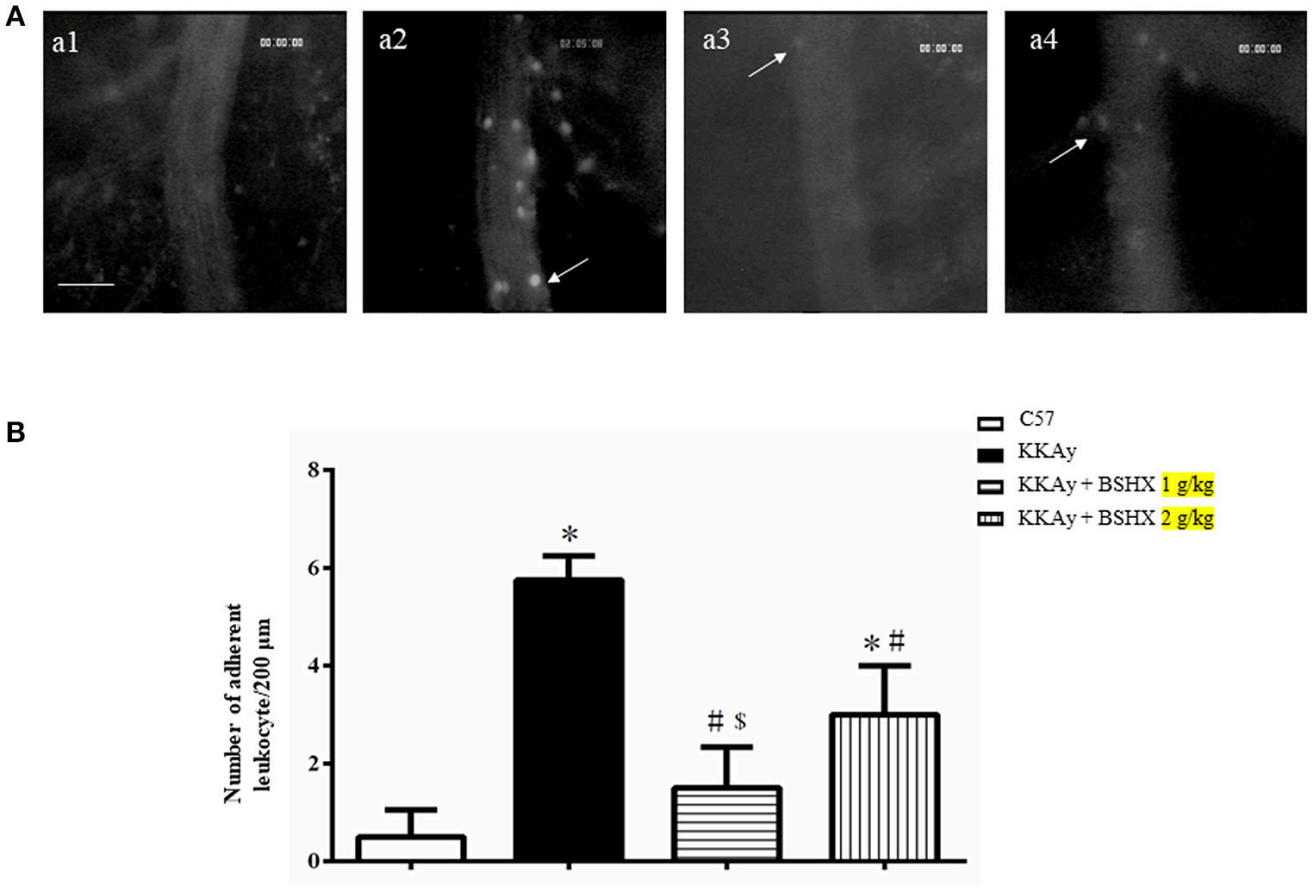
The effect of BSHX on leukocyte adhesion in cerebral venules. **(A)** Representative pictures of leukocyte adhesion in various groups. Arrows indicate adherent leukocytes. a1: C57 group; a2: KKAy group; a3: KKAy + BSHX 1 g/kg group; a4: KKAy + BSHX 2 g/kg. Bar = 50 μm. **(B)** Statistic analysis of the number of leukocytes adherent to venular wall. Data were expressed as mean ± SD (*n* = 6). ^*^p < 0.05 vs. C57 group, ^#^*p* < 0.05 vs. KKAy group, ^$^*p* < 0.05 vs. KKAy+BSHX 2 g/kg group.

Cerebral microcirculation was assessed in terms of albumin leakage and leukocyte adhesion. The albumin leakage in KKAy group increased significantly relative to the control group, which was diminished apparently by BSHX treatment (Figure 4). The benefit of BSHX treatment on cerebral microcirculation disorders in KKAy mice was also evidenced by the attenuation of leukocyte adhesion (Figure 5).

### BSHX increases the number of open microvessels in hippocampus of KKAy mice

**Figure 6:**
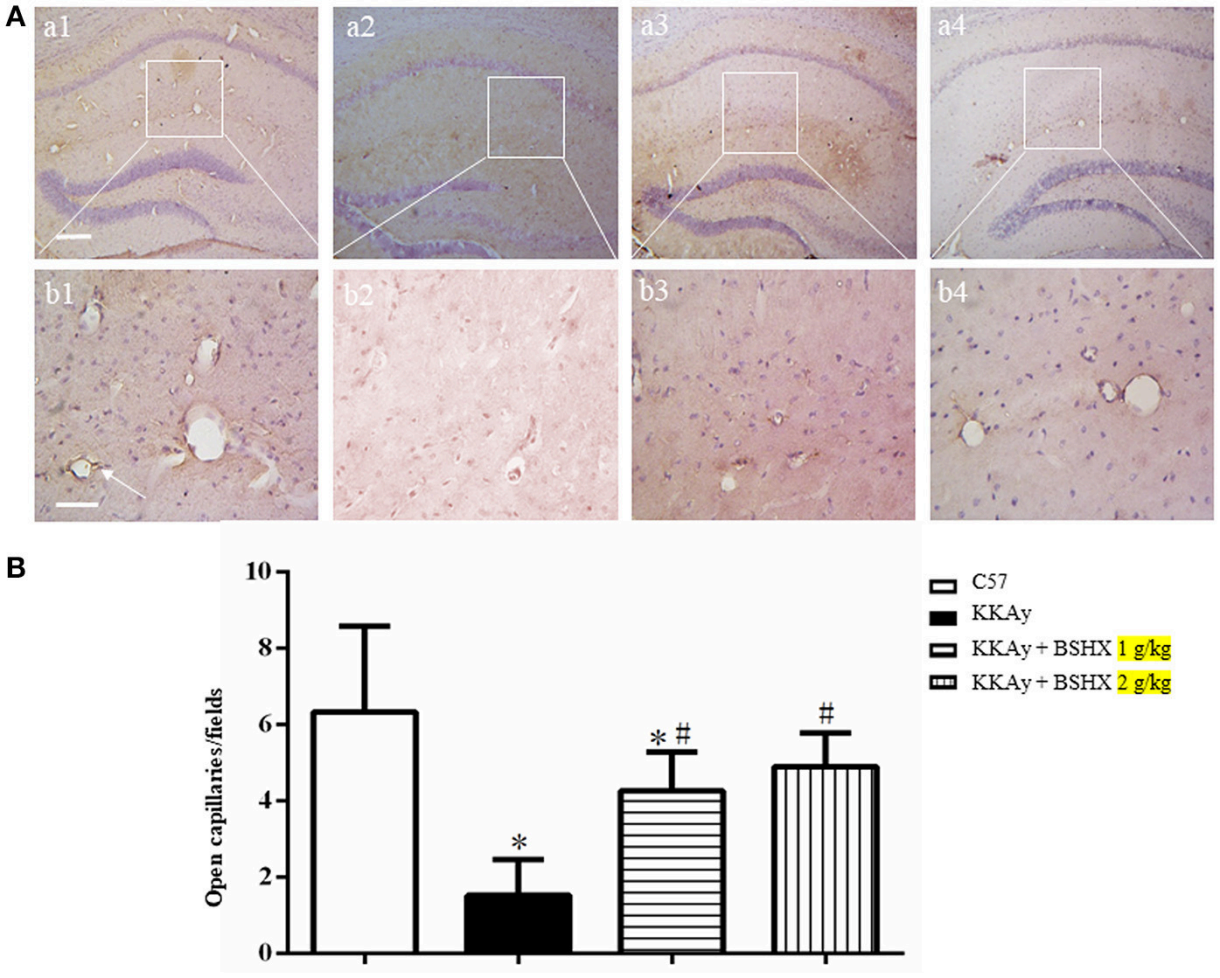
The effect of BSHX on the number of open microvessels in mouse hippocampus. **(A)** Representative immunohistochemistry images of mouse hippocampus region in different groups. Arrows represent open microvessels. a1: C57 group; a2: KKAy group; a3: KKAy + BSHX 1 g/kg group; a4: KKAy + BSHX 2 g/kg. Bar = 200 μm. High magnifications of a1–a4 are shown below as b1–b4, respectively. Bar = 50 μm. **(B)** Quantitative analysis of CD-31 positive opening microvessels. Data were expressed as mean ± SD (*n* = 5). ^*^p < 0.05 vs. C57 group, ^#^*p* < 0.05 vs. KKAy group.

To evaluate the number and morphology of microvessels, an immunochemistry staining for CD31 was performed to delineate the vessels. As depicted in Figure 6A, the number of open microvessels in hippocampal CA1 region was reduced in KKAy group with contracted vasculature and thickening vessel wall. BSHX obviously attenuated the alteration in microvessels in KKAy mice. Figure 6B shows a quantitative evaluation of the number of open microvessels in the CA1 region, confirming the above results.

### BSHX attenuates the alteration in ultrastructure of cerebral microvessels of KKAy mice

**Figure 7:**
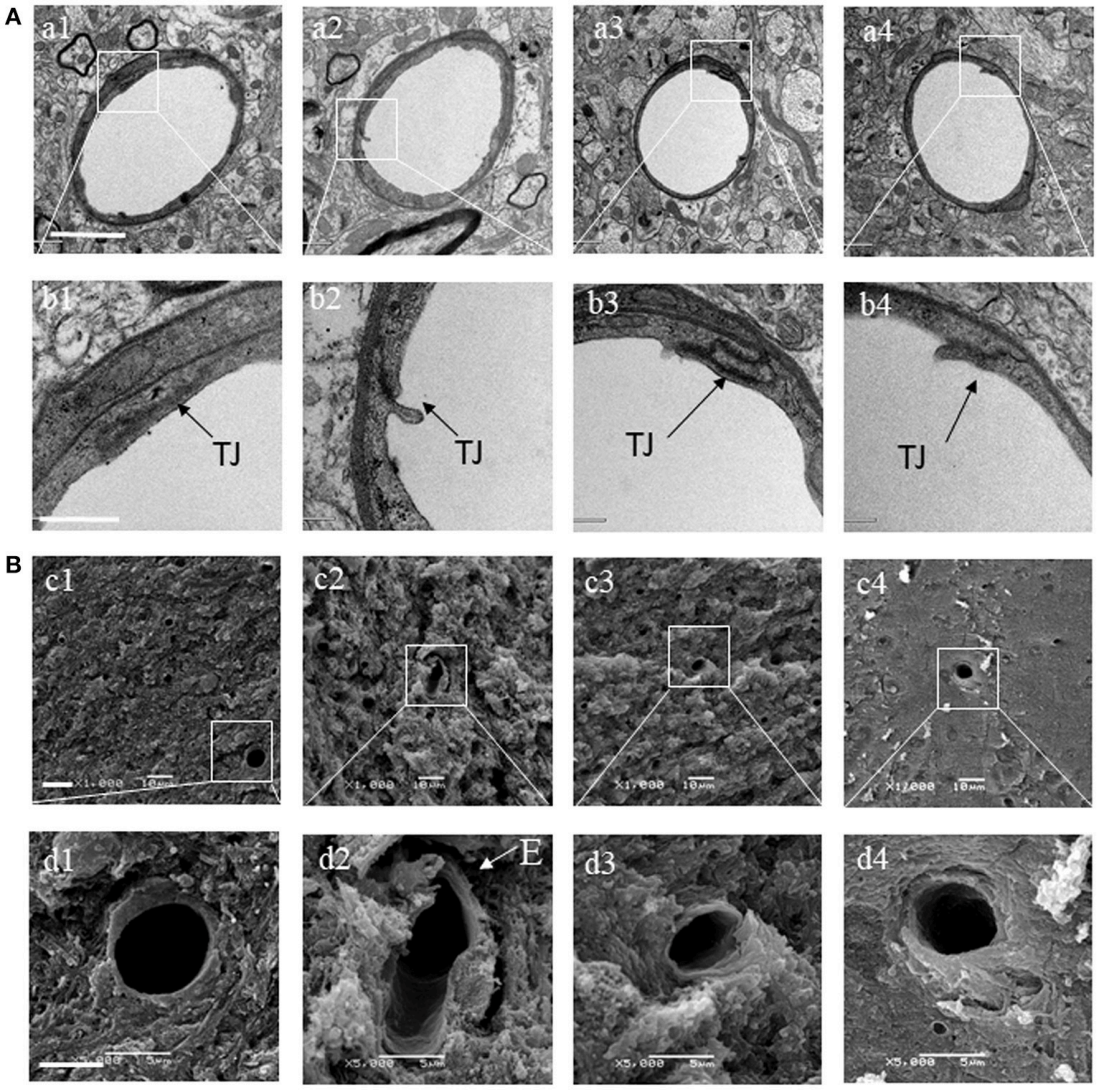
The effect of BSHX on ultrastructure of microvessels in cerebral hippocampal CA1 sector. **(A)** Representative images of the cerebral hippocampus from different groups detected by TEM (upper two panels). **(B)** Representative images of the cerebral hippocampus from different groups detected by SEM (lower two panels). Micrographs in b,d are the high magnification of the area inside the boxes in a,b, respectively. a1: C57 group; a2: KKAy group; a3: KKAy + BSHX 1 g/kg group; a4: KKAy + BSHX 2 g/kg. TJ: tight junction. Bar (a1) = 2 μm; Bar (b1) = 0.5 μm; Bar (c1) = 1 μm; Bar (d1) = 5 μm.

Transmission electron microscopy was performed to examine the microvasculature in the cerebral hippocampal CA1 sector. Compared with the C57 group (Figures 7A a1,b1), the KKAy group showed a remarkable alteration in the microvessels manifested as perivascular edema, indicative of breakdown of endothelial cells barrier (Figures 7A a2,b2). These changes were alleviated by treatment with BSHX (Figures 7A a3,b3,a4,b4). In line with the results observed by TEM, examination by scanning electron microscopy revealed an obvious reduction in the number of open microvessels and an increase in perivascular edema in KKAy group (Figures 7B c2,d2) in comparison with C57 group (Figures 7B c1,d1). BSHX treatment notably alleviated the alterations in cerebral microvasculature, reducing the perivascular edema and increasing the number of open capillaries (Figures 7B c3,d3,c4,d4).

### BSHX alleviates degradation of tight junction proteins in KKAy mouse cerebral tissue

**Figure 8:**
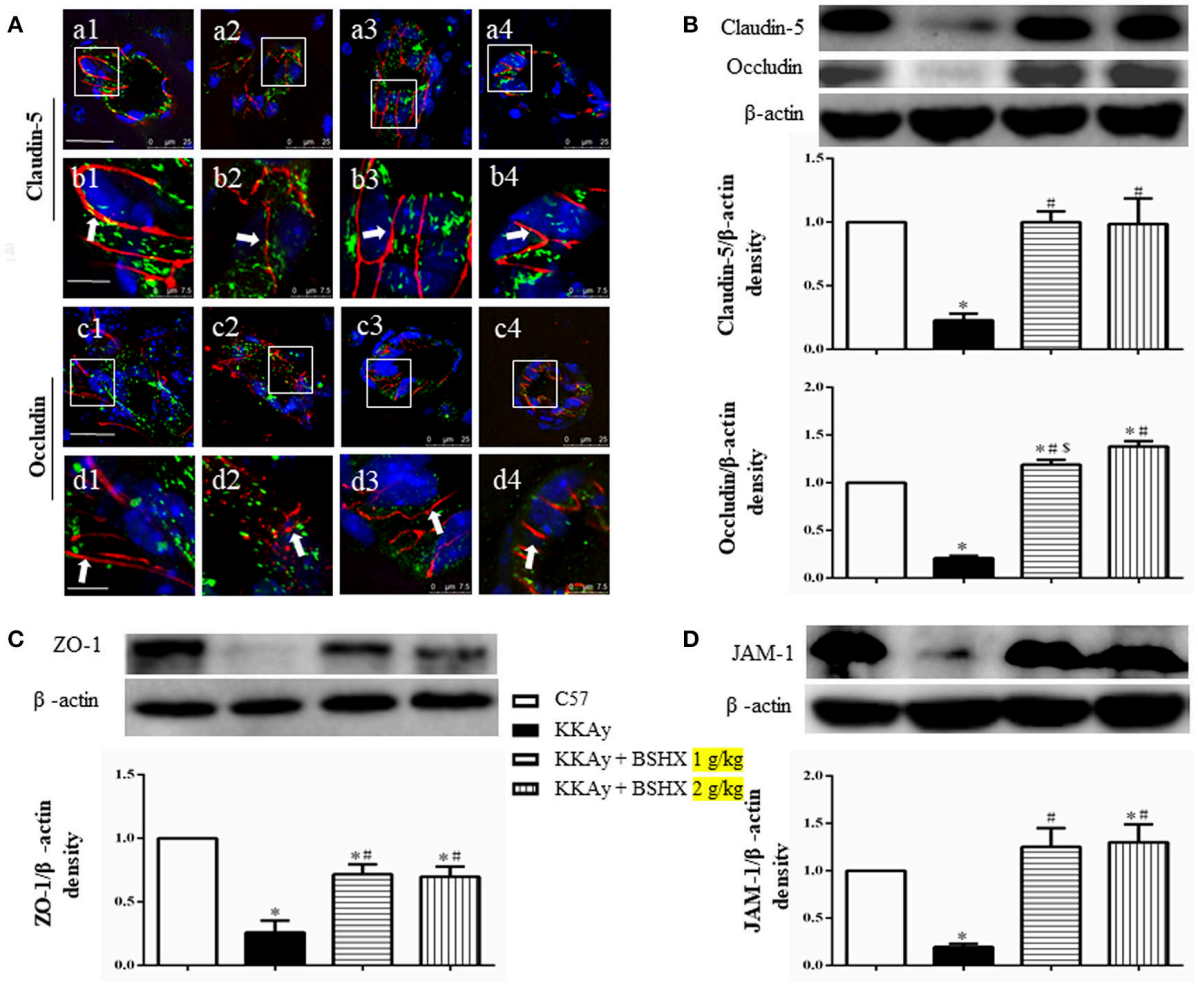
The effect of BSHX on TJ proteins expression in vascular endothelial cells. **(A)** Representative immunofluorescence confocal images of claudin-5 (a1–b4, red), occludin (c1–d4, red) localized at the periphery of endothelial cells with marker vWF (green). Arrows indicate the localization of claudin-5 or occludin. a1: C57 group; a2: KKAy group; a3: KKAy + BSHX 1 g/kg group; a4: KKAy + BSHX 2 g/kg. TJ: tight junction. Bar (a1,c1) = 25 μm; Bar (b1,d1) = 7.5 μm. **(B)** Representative Western blots and quantitative analysis of claudin-5 and occludin. **(C)** Representative western blots and quantitative analysis of ZO-1. **(D)** Representative western blots and quantitative analysis of JAM-1. Data were expressed as mean ± SD (*n* = 5). ^*^p < 0.05 vs. C57 group, ^#^*p* < 0.05 vs. KKAy group, ^$^*p* < 0.05 vs. KKAy+BSHX 2 g/kg group.

The expression of tight junction (TJ) proteins in endothelial cells is crucial for preserving their barrier function (Hirase et al., 1997; Ohtsuki et al., 2007; Luissint et al., 2012). Thus, vascular endothelial TJ proteins in different groups were determined by immunofluorescent staining and western blot. Claudin-5 was detected between endothelial cells as continuous lines in C57 group (Figures 8A a1,b1), while these continuous distributions were disrupted evidently in KKAy group, becoming dotted lines (Figures 8A a2,b2). In addition, a prominent decrease in claudin-5 fluorescence intensity in KKAy group indicated a decrease in the expression of this protein. By contrast, treatment with BSHX inhibited the breakdown of claudin-5 notably (Figures 8A a3,b3,a4,b4). These observations were further verified by western blot (Figure 8B). Likewise, down-regulation of another TJ protein occludin in KKAy mice was observed, which was significantly restored by BSHX treatment (Figures 8A,B).

Similar results were also observed for the expression of JAM-1 and ZO-1, the other two TJ proteins (Figures 8C,D). Together with the results for claudin-5 and occludin, these observations highlight the restoration of TJ proteins as a contributor to the benefit of BSHX in KKAy mice.

### BSHX alleviates neurons damage in CA1 sector of KKAy mice

**Figure 9:**
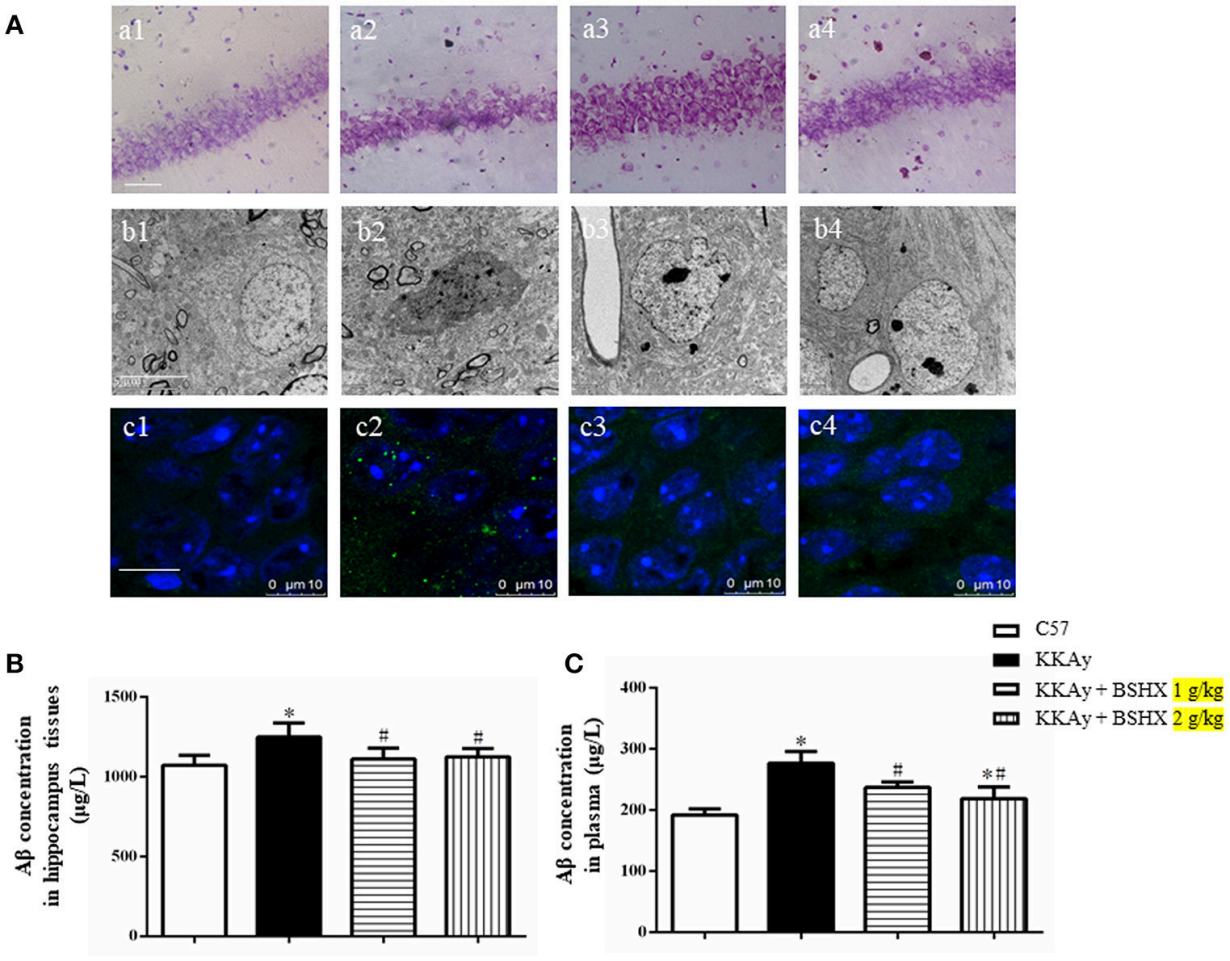
The effect of BSHX on neurons damage in CA1 sector. **(A)** Representative Nissl staining (a), transmission electron micrographs of neurons (b) and immunofluorescence confocal images of Aβ (c) in the cerebral hippocampal CA1 of mice from different groups. 1: C57 group; 2: KKAy group; 3: KKAy + BSHX 1 g/kg group; 4: KKAy + BSHX 2 g/kg. **(B)** The effect of BSHX on the expression levels of Aβ in hippocampus tissues of mice. **(C)** The effect of BSHX on the expression levels of Aβ in plasma of mice. Data were expressed as mean ± SD (*n* = 6). ^*^p < 0.05 vs. C57 group, ^#^*p* < 0.05 vs. KKAy group.

The effect of BSHX on neuron damage in CA1 sector of KKAy mice was first documented by Nissl staining. Neurons were closely arranged in approximately three to four layers and packed regularly with the Nissl bodies being darkly stained in C57 group (Figures 9A a1). In contrast, mice in KKAy group exhibited severe pathological changes characterized by thinning of the cell layers and cell swelling in hippocampal CA1 subfields (Figures 9A a2). BSHX at both dosages significantly attenuated these neuronal damages (Figures 9A a3,a4).

Displayed in Figures 9A b1–b4 are the representative images of hippocampal neurons in different groups observed by TEM. Compared with the neurons in C57 group (Figures 9A b1), marked changes occurred in the ultrastructural feature of hippocampal neurons in KKAy group, such as shrinkage of both nucleus and cytoplasm with increased electron density (Figures 9A b2). Treatment with BSHX (Figures 9A b3,b4) significantly ameliorated these alterations in hippocampal neurons of KKAy mice.

Aβ deposition is known as a histopathological hallmark of cognitive impairment in DM patients and animals. Therefore, we first assessed the effect of BSHX prescription on Aβ in hippocampus of KKAy mice by immunofluorescent staining. As illustrated in Figures 9A c1–c4, treatment with BSHX obviously reduced the accumulation of Aβ in hippocampal CA1 subfields. We further measured the expression of Aβ in hippocampus and serum by using ELISA. Consistently, BSHX treatment significantly alleviated the elevated expression of Aβ in hippocampus tissue (Figure 9B) and plasma (Figure 9C) in KKAy mice.

### BSHX inhibits the expression of AGEs-RAGE and RhoA/ROCK/moesin signaling pathway

**Figure 10:**
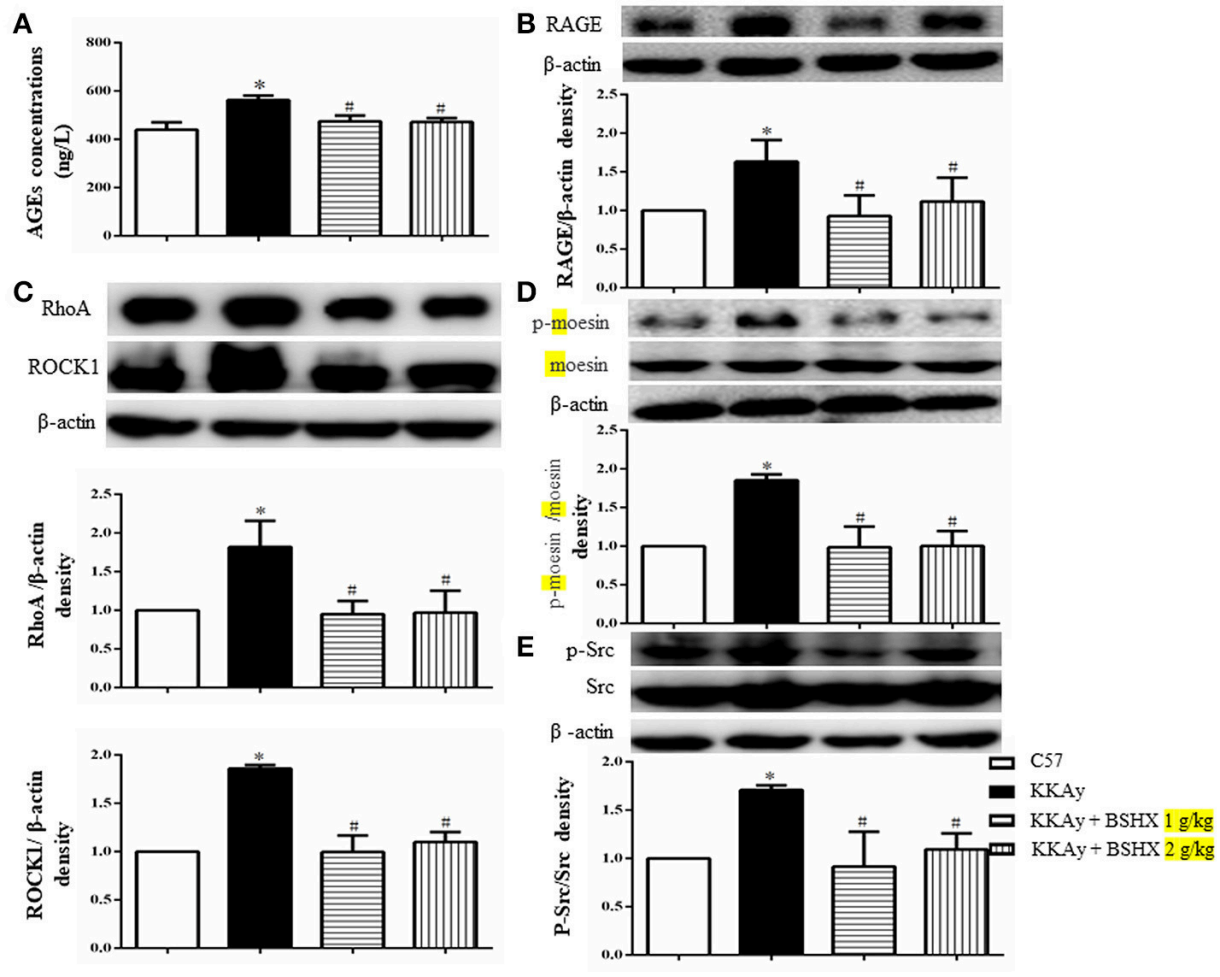
The effect of BSHX on the expression levels of AGE, RAGE, RhoA, ROCK1, p-Mosein and Src phosphorylation. **(A)** Level of AGEs. **(B)** Representative western blots and quantitative analysis of RAGE in various groups. **(C)** Representative western blots and quantitative analysis of RhoA, ROCK1 in various groups. **(D)** Representative western blots and quantitative analysis of moesin and p-moesin in various groups. **(E)** Representative western blots and quantitative analysis of Src and p-Src in various groups. Data were expressed as mean ± SD (*n* = 6). ^*^p < 0.05 vs. C57 group, ^#^*p* < 0.05 vs. KKAy group.

In view of the role of the formation and aggregation of AGEs in the pathogenesis of diabetic microvascular complications (Neviere et al., 2016), we firstly determined the expression levels of AGEs in hippocampus by ELISA. BSHX treatment significantly attenuated the increased expression of AGEs in KKAy mice (Figure 10A). A concurrent alteration occurred in the expression of RAGE as shown by western blot (Figure 10B). The RhoA/ROCK/moesin mediates the AGE action. Thus, western blot was employed to determine whether BSHX is able to down-regulate the expression of RhoA, ROCK1 and p-moesin in KKAy mice. As shown in Figures 10C,D, a significant increase in RhoA, ROCK1 and p-moesin protein levels was observed in KKAy group. Evidently, after 12 weeks of BSHX treatment, the up-regulation of RhoA, ROCK1 and p-moesin was notably inhibited.

Src activation participates in downregulation of TJ proteins, leading to endothelial barrier dysfunction (Adam et al., 2016). Consistently, a remarkable increase in the phosphorylation of Src kinase was observed in KKAy group compared with C57 group. Following treatment with BSHX, there was a significant decrease in the levels of phosphorylation of Src kinase (Figure 10E).

Taken together, these results suggest that BSHX may exert inhibitory effect on the endothelial barrier dysfunction at least partly via inhibiting the expression of RhoA/ROCK/moesin proteins and the phosphorylation of Src kinase.

## Discussion

The present study demonstrated the benefit of BSHX for the cognitive impairment in diabetic KKAy mice, which concomitantly reduced random blood glucose and fasting blood glucose levels, ameliorated cerebral microcirculation dysfunction, attenuated hippocampal neuron injury, and protected Aβ accumulation. These findings suggested BSHX as a potential option for intervention of diabetes-induced cognitive impairment. Moreover, BSHX inhibited AGE-RAGE induced upregulation of RhoA, ROCK, phosphorylation of moesin and Src, highlighting the involvement of this signaling pathway in BSHX action.

As a complication of T2DM, cognitive impairment has received increasing attention because of its impact on the diabetes management and quality of life. The pathogenesis of cognitive impairment in T2DM is not fully understood. Several mechanism has been proposed to account for this complication, including hyperglycemia and hypoglycemia, microvascular dysfunction, and Aβ formation and accumulation, suggesting an involvement of multiple insults in the etiology (Li et al., 2017). As a consequence, any strategy directing at a single insult may not be effective enough, and a remedy targeting multiple links is likely required to treat this complication. The results of the present study suggest that BSHX may function as such a medication.

The multiple targeting potential of BSHX in fighting against T2DM -related cognition dysfunction is predictable if consider the data so far available found for the role of the components contained in this preparation. Of them, *Cuscuta chinensis* Lam has been demonstrated to reduce blood glucose in streptozotocin (STZ) induced diabetic rats (Dao-Zhong et al., 2008; Rath et al., 2016). The anti-hyperglycemia potential was also reported for *Schisandra chinensis* (Turcz.) Baill (Yuan et al., 2002; An et al., 2012). In addition, schisandrin B, an ingredient of *Schisandra chinensis* (Turcz.) Baill, has been found to promote the depolymerization of Aβ oligomers (Yang et al., 2017). Icariin, a flavonoid compound from the herb *Epimedium brevicornu* Maxim, promotes neuronal survival by attenuating Aβ_25−35_-induced tau protein hyperphosphorylation (Zeng et al., 2010). Some components of BSHX were found to prevent microvascular dysfunction in diabetes. For example, *Whitmania pigra* Whitman has shown ability to delay the diabetic microangiopathy (Zhou et al., 2002). Total flavonoids of *Epimedium brevicornu* Maxim demonstrate a protective effect on the vascular endothelial injury in diabetic mice (Han et al., 2011). Most likely, it is the collective action of these components that leads to the benefit role of BSHX in cognitive impairment in KKAy mice.

Among the insults proposed to link with T2DM-related cognitive impairment, diabetes-specific vascular disease has attracted increasing attention (Turnbaugh et al., 2006; Gorelick et al., 2011). The mechanistic study points to the role of AGEs in the progression of vascular complications in diabetes (Guo et al., 2009; Hirose et al., 2010; Zhang et al., 2017). Cumulative hyperglycemic exposure enhances the accumulation of AGEs, which bind to RAGE leading to endothelial barrier disruption and the development and progression of vascular complications in diabetes (Yamagishi et al., 2008a,b). Signaling events downstream of the AGE-RAGE pathway are complex, including the regulation of RhoA/ROCK/moesin pathway and phosphorylation of Src (Guo et al., 2009; Hirose et al., 2010; Zhang et al., 2017). RhoA, a member of the Rho GTPase family, is correlated with various cellular and biological functions such as cytoskeleton regulation and cell development. The Rho kinases (ROCK) are the RhoA effectors with ROCK-1 as the major downstream effector of RhoA. Rho-ROCK signaling is involved in permeability changes under inflammatory conditions (Mong and Wang, 2009; Cui et al., 2017). Up-regulation of RhoA and ROCK contributes to AGE-induced endothelial dysfunction (Wang et al., 2012; Hou et al., 2017). Moesin, belonging to ERM protein family, is the downstream target of RhoA/ROCK pathway. Phosphorylation of moesin is closely associated with cytoskeletal re-arrangement and subsequent endothelial monolayer permeability (Adyshev et al., 2011). Concurrently, binding of AGEs with RAGE enhances the phosphorylation of Src, which down-regulates TJ-associated proteins, resulting in increased endothelial permeability (Basuroy et al., 2003; Sheikpranbabu et al., 2010; Puri and Walker, 2013). Consistent with these reports, we observed a dysfunctional cerebral microcirculation in KKAy mice, manifested by a decreased expression of TJ proteins and breakdown of endothelial barrier. These alterations are associated with an elevation in AGEs and RAGE, as well as up-regulation of RhoA and ROCK, and subsequent activation of moesin and Src. Importantly, all these alterations were significantly attenuated by BSHX, indicating the crucial role of cerebral microvessels in mediating the effect of this medicine. However, it is noteworthy that RhoA has also been reported to enhance barrier function in either basal or S1P-stimulated endothelial cells (Szulcek et al., 2013; Zhang et al., 2016). Likewise, ROCK has been reported to promote lymphatic endothelial barrier function (Breslin, 2011). Besides, thrombin-mediated RhoA activation at the ruffles temporarily enhances the barrier function, whereas RhoA activation at other locations distal to the cell periphery has been shown to be involved in thrombin-mediated disruption of monolayer (Szulcek et al., 2013). These findings suggest that the intracellular location of active RhoA may determine its distinct effects on endothelial permeability (Szulcek et al., 2013). Nevertheless, the causal link between the observed effects of BSHX needs to be identified by further study.

In conclusion, the present study demonstrates that BSHX prescription effectively improves diabetes-induced cognitive impairment and cerebral microangiopathy through inhibition of AGEs-RAGE induced expression of RhoA/ROCK/moesin signaling pathway. These results suggest BSHX as a potential regime to prevent diabetic induced cognitive impairment, and provide novel insight for better understanding the rationale behind its effects.

## Author contributions

YL designed the experiments, acquired data in all Figures and wrote the manuscript. LY(Li Yan), Y-YL, B-HH, LY(Lei Yang) and S-YZ participated in animal experiments. C-SP and PH participated in biomolecular experiment. C-SW, X-MW, J-YF and QL revised the manuscript. J-YH designed the experiments, revised the manuscript, interpreted data and finally approved the submission of this manuscript.

### Conflict of interest statement

The authors declare that the research was conducted in the absence of any commercial or financial relationships that could be construed as a potential conflict of interest.

## Abbreviations

BSHX: Bushen Huoxue
DM: Diabetes mellitus
T2DM: Type 2 Diabetes mellitus
CBF: cerebral blood flow
TJ: tight junction proteins
ZO-1: zonula occluden-1
MWM: Morris Water Maze
AGEs: advanced glycation end products
RAGE: receptor of glycation end products
β-Amyloid: Aβ
SEM: scanning electron microscopy
TEM: transmission electron microscopy.

## References

[B1] Adam A. P. Lowery A. M. Martino N. Alsaffar H. Vincent P. A. (2016). Src family kinases modulate the loss of endothelial barrier function in response to TNF-α: crosstalk with p38 signaling. PLoS ONE 11:e0161975. 10.1371/journal.pone.0161975 27603666 PMC5014308

[B2] Adyshev D. M. Moldobaeva N. K. Elangovan V. R. Garcia J. G. N. Dudek S. M. (2011). Differential involvement of ezrin/radixin/moesin proteins in sphingosine 1-phosphate-induced human pulmonary endothelial cell barrier enhancement. Cell. Signal. 23, 2086–2096. 10.1016/j.cellsig.2011.08.003 21864676 PMC3651873

[B3] An L. P. Wang Y. P. Liu X. M. Wang C. M. Zhan J. Z. Sun H. (2012). Effect of schisandrae fructus oil on type 2 diabetic rats induced by Streptozotocin. China Tradit. Herbal Drugs 43, 552–556.

[B4] Ascher-Svanum H. Chen Y. F. Hake A. Kahle-Wrobleski K. Schuster D. Kendall D. (2015). Cognitive and functional decline in patients with mild alzheimer dementia with or without comorbid diabetes. Clin. Ther. 37, 1195–1205. 10.1016/j.clinthera.2015.01.002 25676448

[B5] Basuroy S. Sheth P. Kuppuswamy D. Balasubramanian S. Ray R. M. Rao R. K. (2003). Expression of kinase-inactive c-Src delays oxidative stress-induced disassembly and accelerates calcium-mediated reassembly of tight junctions in the Caco-2 cell monolayer. J. Biol. Chem. 278, 11916–11924. 10.1074/jbc.M211710200 12547828

[B6] Bingham D. Martin S. J. Macrae I. M. Carswell H. V. (2012). Watermaze performance after middle cerebral artery occlusion in the rat: the role of sensorimotor versus memory impairments. J. Cereb. Blood Flow Metabol. 32, 989–999. 10.1038/jcbfm.2012.16 22373646 PMC3367220

[B7] Breslin J. W. (2011). ROCK and cAMP promote lymphatic endothelial cell barrier integrity and modulate histamine and thrombin-induced barrier dysfunction. Lymphat. Res. Biol. 9, 3–11. 10.1089/lrb.2010.0016 21417762 PMC3060730

[B8] Chen F. Dong R. R. Zhong K. L. Ghosh A. Tang S. S. Long Y.. (2016). Antidiabetic drugs restore abnormal transport of amyloid-β across the blood-brain barrier and memory impairment in db/db mice. Neuropharmacology 101, 123–136. 10.1016/j.neuropharm.2015.07.023 26211973

[B9] Chen T. B. Yiao S. Y. Sun Y. Lee H. J. Yang S. C. Chiu M. J.. (2017). Comorbidity and dementia: a nationwide survey in Taiwan. PLoS ONE 12:e0175475. 10.1371/journal.pone.0175475 28403222 PMC5389824

[B10] Cui Y. C. Pan C. S. Yan L. Li L. Hu B. H. Chang X.. (2017). Ginsenoside Rb1 protects against ischemia/reperfusion-induced myocardial injury via energy metabolism regulation mediated by RhoA signaling pathway. Sci. Rep. 7:44579. 10.1038/srep44579 28327605 PMC5361119

[B11] Dao-Zhong L. I. Peng D. Y. Xian-Xiang X. U. Zhang R. (2008). Research on mechanism of cuscuta chinensis polysaccharide effects on diabetes mellitus. China Arch. Tradit. Chin. Med. 26, 2717–2718. 10.13193/j.archtcm.2008.12.190.lidzh.076

[B12] Fried P. J. Schilberg L. Brem A. K. Saxena S. Wong B. Cypess A. M.. (2016). Humans with Type-2 diabetes show abnormal long-term potentiation-like cortical plasticity associated with verbal learning deficits. J. Alzheimers Dis. 55, 89. 10.3233/JAD-160505 27636847 PMC5193103

[B13] Gorelick P. B. Scuteri A. Black S. E. Decarli C. Greenberg S. M. Iadecola C.. (2011). Vascular contributions to cognitive impairment anddementia: a statement for healthcare professionals from the American HeartAssociation/American Stroke Association. Stroke 42, 2672–2713. 10.1161/STR.0b013e3182299496 21778438 PMC3778669

[B14] Greenberg S. M. Vernooij M. W. Cordonnier C. Viswanathan A. Salman A. S. Warach S.. (2009). Cerebral microbleeds: a guide to detection and interpretation. Lancet Neurol. 8, 165–174. 10.1016/S1474-4422(09)70013-4 19161908 PMC3414436

[B15] Guo X. Wang L. Chen B. Li Q. Wang J. Zhao M.. (2009). ERM protein moesin is phosphorylated by advanced glycation end products and modulates endothelial permeability. Am. J. Physiol. Heart Circ. Physiol. 297, H238–H246. 10.1152/ajpheart.00196.2009 19395553

[B16] Han A. P. Zhang J. Ding X. S. (2011). Effects of total flavonoids of epimedium on vascular function of diabetic mice. J. Nanjing Univ. Tradit. Chin. Med. 27, 243–246. 10.14148/j.issn.1672-0482.2011.03.015

[B17] Herberg L. Coleman D. L. (1977). Laboratory animals exhibiting obesity and diabetes syndromes. Metabol. Clin. Exp. 26, 59–99. 10.1016/0026-0495(77)90128-7 834144

[B18] Hirase T. Staddon J. M. Saitou M. Ando-Akatsuka Y. Itoh M. Furuse M. (1997). Occludin as a possible determinant of tight junction permeability in ECs. J. Cell Sci. 110:1613. 10.1242/jcs.110.14.1603 9247194

[B19] Hirose A. Tanikawa T. Mori H. Okada Y. Tanaka Y. (2010). Advanced glycation end products increase endothelial permeability through the RAGE/Rho signaling pathway. FEBS Lett. 584, 61–66. 10.1016/j.febslet.2009.11.082 19944695

[B20] Hong F. U. Wang X. M. Liu G. X. (2007). The effect of Jiawei Wuzi Yanzong Particle on memory ability and level of serum beta-amyloid protein of mild cognitive impairment patients. China J. Gerontol. 27, 715–717. 10.3969/j.issn.1005-9202.2007.08.005

[B21] Hou B. Qiang G. Zhao Y. Yang X. Chen X. Yan Y.. (2017). Salvianolic acid a protects against diabetic nephropathy through ameliorating glomerular endothelial dysfunction via inhibiting AGE-RAGE signaling. Cell. Physiol. Biochem. 44, 2378–2394. 10.1159/000486154 29262395

[B22] Huang P. Zhou C. M. QinHu Liu Y. Y. Hu B. H. Chang X.. (2012). Cerebralcare Granule® attenuates blood-brain barrier disruption after middle cerebral artery occlusion in rats. Exp. Neurol. 237, 453–463. 10.1016/j.expneurol.2012.07.017 22868201

[B23] Iadecola C. (2013). The pathobiology of vascular dementia. Neuron 80, 844–866. 10.1016/j.neuron.2013.10.008 24267647 PMC3842016

[B24] Iwatsuka H. Shino A. Suzuoki Z. (1970). General survey of diabetic features of yellow KK mice. Endocrinol. Jpn. 17, 23–35. 10.1507/endocrj1954.17.23 5468422

[B25] Li J. Cesari M. Liu F. Dong B. Vellas B. (2016). Effects of diabetes mellitus on cognitive decline in patients with Alzheimer disease: a systematic review. Can. J. Diabet. 41, 114–119. 10.1016/j.jcjd.2016.07.003 27614804

[B26] Li Y. Zeng K. W. Wang X. M. (2017). [Cerebral microangiopathy of diabetes]. China J. Chin. Materia Med. 42, 2247–2253. 10.19540/j.cnki.cjcmm.2017.0104 28822176

[B27] Luissint A. C. Artus C. Glacial F. Ganeshamoorthy K. Couraud P. O. (2012). Tight junctions at the blood brain barrier: physiological architecture and disease-associated dysregulation. Fluids Barriers CNS 9:23. 10.1186/2045-8118-9-23 23140302 PMC3542074

[B28] Mong P. Y. Wang Q. (2009). Activation of Rho kinase isoforms in lung endothelial cells during inflammation. J. Immunol. 182, 2385–2394. 10.4049/jimmunol.0802811 19201893

[B29] Neviere R. Yu Y. Wang L. Tessier F. Boulanger E. (2016). Implication of advanced glycation end products (Ages) and their receptor (Rage) on myocardial contractile and mitochondrial functions. Glycoconj. J. 33, 1–11. 10.1007/s10719-016-9679-x 27277623

[B30] Ohtsuki S. Sato S. Yamaguchi H. Kamoi M. Asashima T. Terasaki T.. (2007). Exogenous expression of claudin-5 induces barrier properties in cultured rat brain capillary endothelial cells. J. Cell Physiol. 210, 81–86. 10.1002/jcp.20823 16998798

[B31] Prasad S. Sajja R. K. Naik P. Cucullo L. (2014). Diabetes mellitus and blood-brain barrier dysfunction: an overview. J. Pharmacovigil. 2, 125. 10.4172/2329-6887.1000125 25632404 PMC4306190

[B32] Puri P. Walker W. H. (2013). The tyrosine phosphatase SHP2 regulates Sertoli cell junction complexes. Biol. Reprod. 88, 59. 10.1095/biolreprod.112.104414 23325809

[B33] Quaegebeur A. Lange C. Carmeliet P. (2011). The neurovascular link in health and disease: molecular mechanisms and therapeutic implications. Neuron 71, 406–424. 10.1016/j.neuron.2011.07.013 21835339

[B34] Rath D. Kar D. M. Panigrahi S. K. Maharana L. (2016). Antidiabetic effects of Cuscuta reflexa Roxb. in streptozotocin induced diabetic rats. J. Ethnopharmacol. 192, 442–449. 10.1016/j.jep.2016.09.026 27649679

[B35] Roy S. Kim N. Desai A. Komaragiri M. Baxi N. Jassil N.. (2015). Cognitive function and control of Type 2 diabetes mellitus in young adults. N. Am. J. Med. Sci. 7, 220–226. 10.4103/1947-2714.157627 26110134 PMC4462818

[B36] Sakata A. Mogi M. Iwanami J. Tsukuda K. Min L. J. Jing F.. (2010). Female exhibited severe cognitive impairment in type 2 diabetes mellitus mice. Life Sci. 86, 638–645. 10.1016/j.lfs.2010.03.003 20226793

[B37] Sheikpranbabu S. Kalishwaralal K. Lee K. J. Vaidyanathan R. Eom S. H. Gurunathan S. (2010). The inhibition of advanced glycation end-products. Biomaterials 31, 2260–2271. 10.1016/j.biomaterials.2009.11.076 19963272

[B38] Song X. Wang W. Kang Y. Zhang X. Jiang Y. Yue Z.. (2015). Tangzhining exhibits a protective effect against cognitive dysfunction in diabetic rats. Int. J. Clin. Exp. Med. 8, 9013–9021. 26309554 PMC4538009

[B39] Spampinato S. F. Merlo S. Sano Y. Kanda T. Sortino M. A. (2017). Astrocytes contribute to Aβ-induced blood brain barrier damage through activation of endothelial MMP9. J. Neurochem. 142, 464–477. 10.1111/jnc.14068 28488764

[B40] Strachan M. W. (2011). R D Lawrence Lecture 2010. The brain as a target organ in Type 2 diabetes: exploring the links with cognitive impairment and dementia. Diabet. Med. 28, 141–147. 10.1111/j.1464-5491.2010.03199.x 21219420

[B41] Sun K. Hu Q. Zhou C. M. Xu X. S. Wang F. Hu B. H.. (2010). Cerebralcare Granule, a Chinese herb compound preparation, improves cerebral microcirculatory disorder and hippocampal CA1 neuron injury in gerbils after ischemia-reperfusion. J. Ethnopharmacol. 130, 398–406. 10.1016/j.jep.2010.05.030 20580803

[B42] Szulcek R. Beckers C. M. Hodzic J. De W. J. Chen Z. Grob T.. (2013). Localized RhoA GTPase activity regulates dynamics of endothelial monolayer integrity. Cardiovasc. Res. 99, 471–482. 10.1093/cvr/cvt075 23536606 PMC3841417

[B43] Talbot K. Wang H. Y. Kazi H. Han L. Y. Bakshi K. P. Stucky A.. (2012). Demonstrated brain insulin resistance in Alzheimer's disease patients is associated with IGF-1 resistance, IRS-1 dysregulation, and cognitive decline. J. Clin. Invest. 122, 1316–1338. 10.1172/JCI59903 22476197 PMC3314463

[B44] Tomino Y. (2012). Lessons from the KK-Ay mouse, a spontaneous animal model for the treatment of human Type 2 diabetic Nephropathy 4, 524–529. 10.5812/numonthly.1954 23573479 PMC3614295

[B45] Turnbaugh P. J. Ley R. E. Mahowald M. A. Magrini V. Mardis E. R. Gordon J. I. (2006). An obesity-associated gut microbiome with increased capacity for energy harvest. Nature 444:1027. 10.1038/nature05414 17183312

[B46] Viswanathan A. Rocca W. A. Tzourio C. (2009). Vascular risk factors and dementia How to move forward? Neurology 73, 1934–1935. 10.1212/01.wnl.0000341271 19171835 PMC2677504

[B47] Wang Q. Fan A. Yuan Y. Chen L. Guo X. Huang X.. (2016). Role of Moesin in Advanced Glycation End Products-Induced Angiogenesis of Human Umbilical Vein Endothelial Cells. Sci. Rep. 6:22749. 10.1038/srep22749 26956714 PMC4783699

[B48] Wang J. Liu H. Chen B. Li Q. Huang X. Wang L.. (2012). RhoA/ROCK-dependent moesin phosphorylation regulates AGE-induced endothelial cellular response. Cardiovasc. Diabetol. 11, 7. 10.1186/1475-2840-11-7 22251897 PMC3280169

[B49] Yamagishi S. Nakamura K. Matsui T. Noda Y. Imaizumi T. (2008a). Receptor for advanced glycation end products (RAGE): a novel therapeutic target for diabetic vascular complication. Curr. Pharm. Des. 14, 487–495. 10.2174/138161208783597416 18289075

[B50] Yamagishi S. Nakamura K. Matsui T. Ueda S. Fukami K. Okuda S. (2008b). Agents that block advanced glycation end product (AGE)-RAGE (receptor for AGEs)-oxidative stress system: a novel therapeutic strategy for diabetic vascular complications. Expert Opin. Investig. Drugs 17, 983–996. 10.1517/13543784.17.7.983 18549336

[B51] Yamagishi S. Nakamura N. Suematsu M. Kaseda K. Matsui T. (2015). Advanced glycation end products: a molecular target for vascular complications in diabetes. Mol. Med. 21(Suppl. 1):S32. 10.2119/molmed.2015.00067 26605646 PMC4661053

[B52] Yan B. Y. Pan C. S. Mao X. W. Yang L. Liu Y. Y. Yan L.. (2014). Icariside II improves cerebral microcirculatory disturbance and alleviates hippocampal injury in gerbils after ischemia-reperfusion. Brain Res. 1573 63–73. 10.1016/j.brainres.2014.05.023 24858929

[B53] Yang Q. Na L. I. Sui X. Xiao-Hua L. I. Shi X. Z. Liu Y. N. (2017). Study on neuroprotective effect of schisandrin B by regulating the Aβ and its downstream NF-κB/TNF-α pathway. China J. Tradit. Chin. Med. Pharm. 32, 2064–2069.

[B54] Yuan H. B. Shen Z. M. Yin J. W. Lin feng XU. (2002). The hypoglycemic effect of α-glucosidase inhibitor separated from Schizandra chinensis. China J. Biochem. Pharm. 23, 112–114. 10.3969/j.issn.1005-1678.2002.03.002

[B55] Zeng K. W. Ko H. Yang H. O. Wang X. M. (2010). Icariin attenuates β-amyloid-induced neurotoxicity by inhibition of tau protein hyperphosphorylation in PC12 cells. Neuropharmacology 59, 542–550. 10.1016/j.neuropharm.2010.07.020 20708632

[B56] Zhang W. J. Li P. X. Guo X. H. Huang Q. B. (2017). Role of moesin, Src and ROS in advanced glycation end product-induced vascular endothelial dysfunction. Microcirculation 24:e12358. 10.1111/micc.12358 28129474

[B57] Zhang X. E. Adderley S. P. Breslin J. W. (2016). Activation of RhoA, but Not Rac1, mediates early stages of S1P-induced endothelial barrier enhancement. PLoS ONE 11:e0155490. 10.1371/journal.pone.0155490 27187066 PMC4871357

[B58] Zhou S. P. Tong X. L. Pan L. (2002). Influence of leech on the morphologic changes of retinal microvessel in diabetic rats. J. Tradit. China Ophthalmol. 12, 79–82. 10.3969/j.issn.1002-4379.2002.02.007

[B59] Zlokovic B. V. (2008). The blood-brain barrier in health and chronic neurodegenerative disorders. Neuron 57, 178–201. 10.1016/j.neuron.2008.01.003 18215617

